# Nervous and Mental Diseases

**Published:** 1904-03

**Authors:** 


NERVOUS AND MENTAL DISEASES. By Archibald Church, M. D., Professor of Nervous and Mental Diseases and Head of Neurological Department, Northwestern University Medical School; and Frederick Peterson, M. D., President New York State Commissioner in Lunacy; Chief of Clinic, Department of Nervous Diseases, College of Physicians and Surgeons, New York. Fourth edition, thoroughly revised and enlarged. Handsome octavo volume of 922 pages, with 338 illustrations. Philadelphia, New York, London: W. B. Saunders & Company. 1903. Cloth, $5.00 net; sheep or half Morocco, $6.00 net.

In this the fourth edition of this work a large part of the text has been re-written, and we are happy to welcome the advent of considerable new matter. The recent fruitful investigations on the subject of nerve healing are thoroughly reviewed, the relation of Herpes zoster to lesions of the posterior root ganglia firmly established, and the combination disease, so-called, that form of epilepsy marked by myoclonus, fully described. Symptomatology and diagnosis has been added to largely, particularly the value of astereagnosis and of Kernig's sign. The insanity department of the work contains a new section prepared by Dr. Adolph Meyer.

The book is equally adapted to the needs of the student or the general practitioner. J. M. L.

